# Thrombus Histology of Basilar Artery Occlusions: Are There Differences to the Anterior Circulation?

**DOI:** 10.1007/s00062-020-00964-5

**Published:** 2020-10-02

**Authors:** M. Berndt, H. Poppert, K. Steiger, J. Pelisek, P. Oberdieck, C. Maegerlein, C. Zimmer, S. Wunderlich, B. Friedrich, T. Boeckh-Behrens, B. Ikenberg

**Affiliations:** 1 Department of Diagnostic and Interventional Neuroradiology, School of Medicine, Technical University of Munich, Munich, Germany; 2 Helios Klinikum München West, Munich, Germany; 3 Institute of Pathology, School of Medicine, Technical University of Munich, Munich, Germany; 4 Department of Vascular and Endovascular Surgery, Klinikum rechts der School of Medicine, Technical University of Munich, Munich, Germany; 5 Department of Vascular Surgery, University Hospital Zurich, Zurich, Switzerland; 6 Klinikum Neuperlach, Munich, Germany; 7 Department of Neurology, Klinikum rechts der Isar, School of Medicine, Technical University of Munich, Munich, Germany

**Keywords:** Stroke, Thrombectomy, Histology, Clot

## Abstract

**Background:**

For patients with acute vessel occlusions of the anterior circulation histopathology of retrieved cerebral thrombi has been reported to be associated to stroke etiology. Due to the relatively small incidence of posterior circulation stroke, exclusive histopathologic analyses are missing for this subgroup. The aim of the study was to investigate thrombus histology for patients with basilar artery occlusions and uncover differences to anterior circulation clots with respect to underlying etiology.

**Methods:**

A total of 59 basilar thrombi were collected during intracranial mechanical recanalization and quantitatively analyzed in terms of their relative fractions of the main constituents, e.g. fibrin/platelets (F/P), red (RBC) and white blood cells (WBC). Data were compared to histopathological analyses of 122 thrombi of the anterior circulation with respect to underlying pathogenesis.

**Results:**

The composition of basilar thrombi differed significantly to thrombi of the anterior circulation with an overall higher RBC amount (median fraction in % (interquartile range):0.48 (0.37–0.69) vs. 0.37 (0.28–0.50), *p* < 0.001) and lower F/P count (0.45 (0.21–0.58) vs. 0.57 (0.44–0.66), *p* < 0.001). Basilar thrombi composition did not differ between the different etiological stroke subgroups.

**Conclusion:**

The results depict a differing thrombus composition of basilar thrombi in comparison to anterior circulation clots with an overall higher amount of RBC. This may reflect different pathophysiologic processes between anterior and posterior circulation thrombogenesis, e.g. a larger proportion of appositional thrombus growth in the posterior circulation.

## Introduction

Basilar artery occlusions (BAO) account for about 1% of all strokes. Importantly, they are associated with high mortality and morbidity rates without treatment [1–4]. As these rates can be reduced dramatically by mechanical thrombectomy (MT), MT is now standard care in most stroke centers [5–8] despite a lack of evidence by large randomized trials. Outcome rates seem to be comparable to those of large vessel occlusions of the anterior circulation [4, 9].

MT allows collection of thrombus material that can subsequently be used for histopathologic analysis. Due to their prevalent occurrence this has been applied in recent years predominantly for thrombi of the anterior circulation. With overall sample sizes of 20 to almost 200 thrombi, several single center studies included only up to 15 thrombi of the posterior circulation [10–15]. This small sample size may explain why no significant differences in thrombus composition were found between anterior and posterior circulation so far [10].

From a pathophysiological point of view it seems mandatory to study thrombi from patients with basilar artery occlusion (basilar thrombi) and those of the anterior circulation separately, as underlying pathogenesis with higher numbers of in situ thrombosis as well as flow conditions are different [16]. It seems plausible that these different conditions also influence thrombus evolution. Thrombus evolution directly affects thrombus composition, which in turn is influenced by local anatomical and flow conditions. Known associations between thrombus histology and underlying pathology or stroke etiology as well as angiographic and clinical outcome [10, 17] are probably not easy to apply to thrombi of the posterior circulation. Therefore, a dedicated histopathological analysis of vertebrobasilar thrombi is warranted.

Aim of the present study was to analyze a collective of basilar thrombi and compare their histopathologic composition to that of anterior circulation thrombi. Differences were further examined with respect to underlying etiology of stroke.

## Material and Methods

As primary end point, basilar thrombi were collected and analyzed in terms of their histopathologic composition. Existing data of a large collective of anterior circulation thrombi were used to compare their composition to basilar thrombi under consideration of the underlying stroke etiology. Clinical and angiographic data of patients with BAO were analyzed based on a prospectively collected database, and data were put into relation to thrombus composition.

The local ethics committee gave the project a positive vote under number 5518/12. If possible, informed consent of the patients was obtained. If patients were unable to decide concerning the informed consent, a waiver of consent was granted by the ethics committee.

### Study Population

At our single comprehensive stroke center, we screened for patients with acute BAO, who were consecutively treated with second generation thrombectomy devices between 2008 and 2017 (*n* = 134). Institutional eligibility criteria for mechanical thrombectomy in BAO as well as technical details of recanalization procedure can be found in [18]. In parts, clinical and neuroradiological parameters of this population were already described in [18]. Of this collective, 59 thrombi (44%) could be gathered and were available for further histological analysis.

The prospectively collected clinical and imaging data were retrospectively analyzed. Basic demographic, clinical, and interventional data of patients were gathered. The National Institutes of Health Stroke Scale (NIHSS) score was assessed by NIHSS-certified neurologists at time of admission and at time of discharge. The modified Rankin Scale (mRS) was used to assess disability at discharge. The modified thrombolysis in cerebral infarction (mTICI) score [19] was determined by two experienced neurointerventionalists in consensus.

Stroke pathogeneses were determined according to the international TOAST (Trial of ORG 10172 in Acute Stroke Treatment) classification [20] on the basis of diagnostic and clinical information available for each patient, including cerebral computed tomography (CT), CT angiography and magnetic resonance imaging, transcranial and extracranial duplex sonography, coagulation tests, long-term electrocardiography recording, and transthoracic or transesophageal echocardiography [21].

Concerning anterior circulation stroke, histological, etiological, clinical and angiographic data were taken from an existing database of 122 large vessel occlusions of the anterior circulation. In this study at the same single comprehensive stroke center in total 137 thrombi were analyzed, including 122 of the anterior circulation [10].

### Histological Analysis of Thrombus Material

**Fig. 1:**
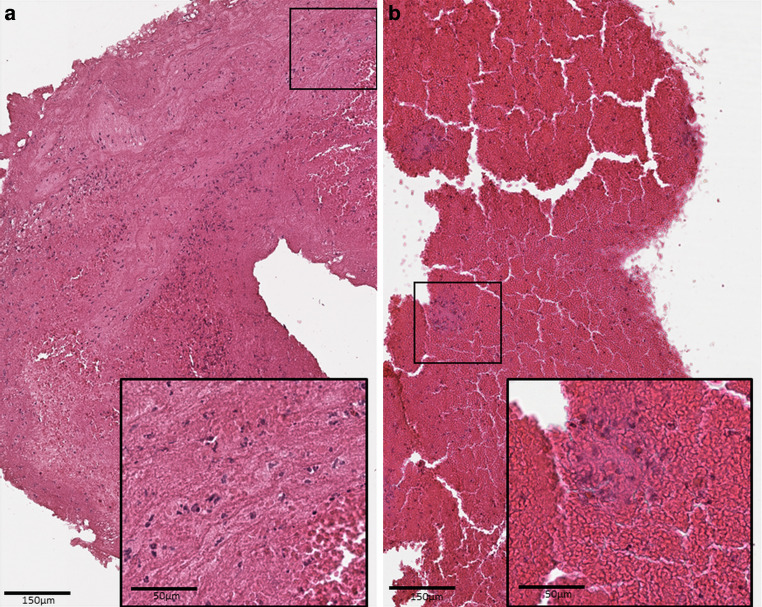
Histopathologic morphology of two hematoxylin-eosin (HE) stained cerebral thrombi. **a** Thrombus retrieved from the middle cerebral artery. **b** thrombus from the basilar artery. Etiology of stroke of each was cardioembolic origin. Comparison of clot fractions depicts higher red blood cell count in the thrombus from posterior circulation. Both thrombi are HE-stained, depicting red blood cells (*red*), white blood cell aggregations (*dark blue*) and fibrin/platelet area (*purple*). *Black bar* 150 μm in the overview image and 50 µm in the small box

All thrombi were processed as previously described [21]: thrombus material was immediately fixed in phosphate-buffered 10% formalin or 3.8% formaldehyde, transferred to 70% ethanol, and then embedded in paraffin. The formalin-fixed and paraffin-embedded thrombus material was cut into 2‑μm slices using a Microm HM 335 E microtome (Microm International GmbH, Walldorf, Germany), followed by hematoxylin-eosin staining of slices. The slides where digitalized at high resolution (0.252 µm per pixel, apparent magnification equivalent to 40× objective) with a Leica AT2 scanning system (Leica, Wetzlar, Germany) and saved as tif-files with Lempel-Ziv-Welch (LZW) compression. Histological analysis of thrombi was performed blinded to clinical and interventional data. The relative quantitative fraction of the different clot components, fibrin/platelets (F/P), red blood cells (RBCs), and white blood cells (WBCs) was evaluated using custom-made quantification software (CAMPThrombus 1.0, not commercially available) of the scanned slides of the complete retrieved thrombus material as reported before [22] (see Fig. 1). In the presence of multiple fragments, all fragments were included in the relative quantitative fraction analysis to ensure the entire clot is represented in the analysis.

Because the number of WBC inside the clots was low compared with the main components F/P and RBC [10], the respective amounts of these two components are approximately inversely proportional. It therefore seems reasonable to take the ratio of RBC**/**F/P (named composition ratio in the following) as an indicator of the overall clot composition.

### Statistical Analysis

Quantitative histological data of thrombi were compared between the groups of anterior and posterior circulation as well as between etiological subgroups by means of nonparametric tests (Wilcoxon rank-sum tests).

*P*-values less than 5% were considered as statistically significant. All statistical analyses were performed using IBM SPSS Statistics (version 25, IBM Corp, Armonk, NY, USA).

## Results

### Patient Characteristics

In total, thrombi from 59 patients with BAO were included in the study. Demographic, clinical and interventional data of patients are presented in Table 1. Histological composition of the basilar thrombi was compared to that of 122 large-vessel occlusions of the anterior circulation (anterior thrombi). Detailed description of this study cohort can be found in [10].

**Table 1:** Patient characteristics. Baseline demographic, clinical and interventional data for all patients with acute basilar artery occlusion (BAO, *n* = 59) and with large vessel occlusion of the anterior circulation (*n* = 122). P‑values of univariate analyses to test for a group difference (t-test for parametric variables, Wilcoxon rank-sum tests for non-parametric variables, Fisher’s exact test for dichotomous categorical variables)

Characteristics	BAO (*n* = 59)	Large vessel occlusions of the anterior circulation (*n* = 122)	Univariate analyses group difference *p*-value
Age, years, median (IQR)	74 (59–81)	72 (60–80)	0.69
Sex, *n* (%) (female)	19 (32%)	61 (50%)	0.03*
mTICI score post recanalization (*n*)	–	–	0.08
0–2a	1	13	–
2b	20	44	–
3	38	65	–
Recanalization time (min, median/IQR)	55 (35–85)	65.5 (28.5–107.3)	0.93
Preinterventional intravenous tPA (*n*)	23	79	0.001*
NIHSS (points, median/IQR)	–	–	–
Pretreatment	15 (9.25–22)	15 (11–18)	0.44
Posttreatment	5 (2–20.25)	6 (1–14)	0.10
mRS score posttreatment (*n*)	*n* = 44	*n* = 60	0.18
good clinical outcome^a^	15	23	–
bad clinical outcome^b^	29	37	–
TOAST	–	–	0.70
Large-artery atherosclerosis (TOAST 1)	11	21	–
Cardioembolic (TOAST 2)	28	58	–
Other determined etiology (TOAST 4)	7	11	–
Other undetermined etiology (TOAST 5)	13	32	–

*mRS* modified Rankin Scale, *NIHSS* National Institute of Health Stroke Scale, *BAO* basilar artery occlusion, *mTICI* modified thrombolysis in cerebral infarction, *TOAST* Trial of ORG 10172 in Acute Stroke Treatment, *tPA* tissue plasminogen activator

*Asterisk* *p* < 0.05

^a^ mRS 0-3 for posterior and mRS 0-2 for anterior circulation

^b^ mRS > 3 for posterior and mRS > 2 for anterior circulation

### Comparison of Thrombus Composition Between Anterior and Basilar Thrombi

**Fig. 2:**
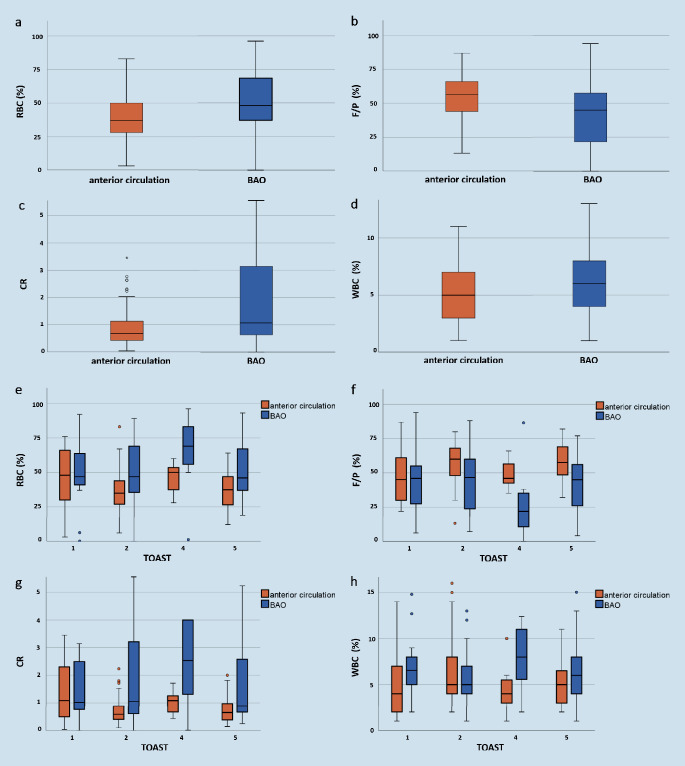
**a–d** Distributions of thrombus compositions for thrombi of the anterior circulation in comparison to basilar thrombi (**a** RBC, **b** F/P, **c** CR, **d** WBC). **e–h** Thrombus composition according to stroke pathogeneses for anterior and basilar thrombi (**e** RBC, **f** F/P, **g** CR, **h** WBC) according to the TOAST classification (*1* large-artery atherosclerosis, *2* cardioembolic, *4* other determined etiology, *5* undetermined etiology). *BAO* basilar artery occlusion, *RBC* Red Blood Cells, *WBC* White Blood Cells, *F/P* Fibrin/Platelets, *CR* Composition Ratio (RBC/FP), TOAST Trial of ORG 10172 in Acute Stroke Treatment

The number of RBC in basilar thrombi (median fraction in % (IQR): 0.48 (0.37–0.69)) was significantly higher than that of anterior circulation thrombi (0.37 (0.28‑0.50), *p* < 0.001). In contrast, BAO thrombi showed significantly lower F/P amount (fraction in %: 0.45 (0.21–0.58) vs. 0.57 (0.44–0.66) for the anterior circulation thrombi, *p* < 0.001). No significant differences were found for WBC components (fraction in %: 0.06 (0.04–0.08) in comparison to thrombi of the anterior circulation (0.05 (0.03‑0.07), *p* = 0.11)). The composition ratio of the two main components (RBC and F/P) differed significantly between basilar (1.07 (0.63–3.14)) and anterior thrombi (0.67 (0.42‑1.14), *p* < 0.001). An overview about relative compositions of basilar and anterior thrombi is shown in Fig. 2a–d.

Thrombus composition of basilar and anterior thrombi according to underlying stroke etiology is displayed in Fig. 2e–h. Corresponding values can be found in Table 2. In patients with LAA stroke (TOAST 1) thrombus composition did not statistically differ between the anterior and posterior circulation (Table 2). For all other stroke subtypes, basilar thrombi showed significantly higher amounts of RBC and composition ratio and lower F/P-proportions than anterior thrombi (for statistical details see Table 2).

**Table 2:** Thrombus composition according to stroke etiology compared between anterior and posterior circulation thrombi. Compositions of thrombi (median fraction in %/IQR) for basilar artery occlusions (BAO, *n* = 59) and for large vessel occlusions of the anterior circulation (*n* = 122) in dependency of their stroke subtype. P‑values of group comparisons (Wilcoxon rank-sum tests) are displayed

Stroke subtype		BAO *n* = 59	Thrombus components (median fraction in %/IQR)	LVOs of the anterior circulation *n* = 122	Thrombus components (median fraction in %/IQR)	Group comparison *p*
Large-artery atherosclerosis (TOAST 1)	RBC	11	0.47/0.37–0.92	21	0.48/0.30–0.72	0.87
	F/P		0.46/0.23–0.94		0.45/0.30–0.82	0.62
	CR		1.02/0.62–15.3		1.09/0.49–2.77	0.67
	WBC		0.07/0.04–0.15		0.04/0.02–0.10	0.16
Cardioembolic (TOAST 2)	RBC	28	0.47/0.36–0.89	57	0.35/0.27–0.63	0.003^*^
	F/P		0.46/0.24–0.80		0.60/0.48–0.78	0.009^*^
	CR		1.05/0.61–10.5		0.58/0.40–1.80	0.004^*^
	WBC		0.05/0.04–0.12		0.05/0.04–0.19	0.44
Other determined etiology (TOAST 4)	RBC	7	0.69/0.50–0.96	11	0.50/0.37–0.60	0.05^*^
	F/P		0.22/0.02–0.87		0.46/0.42–0.66	0.02^*^
	CR		2.54/1.32–48		1.09/0.67–1.71	0.04^*^
	WBC		0.08/0.05–0.12		0.04/0.03–0.10	0.04^*^
Undetermined etiology (TOAST 5)	RBC	13	0.46/0.37–0.93	32	0.38/0.27–0.60	0.04^*^
	F/P		0.45/0.26–0.77		0.58/0.49–0.74	0.02^*^
	CR		0.89/0.66–23.3		0.65/0.38–1.82	0.02^*^
	WBC		0.06/0.04–0.19		0.05/0.03–0.10	0.14

*BAO* basilar artery occlusion, *LVO* large vessel occlusion, *TOAST* Trial of ORG 10172 in Acute Stroke Treatment, *RBC* Red Blood Cells, WBC White Blood Cells, *F/P* Fibrin/Platelets, *CR* Composition Ratio (RBC/FP)

*Asterisk* *p* < 0.05

### Comparison of Thrombus Composition Between Stroke Subtypes Within Anterior and Basilar Thrombi

In patients with basilar artery occlusion, thrombus compositions showed similar RBC and F/P proportions for cardioembolic (TOAST 2) and cryptogenic (TOAST 5) stroke etiologies. Accordingly, values of the composition ratio did not differ significantly between these thrombi (*p* = 0.64). Cardioembolic thrombi (TOAST 2) of the posterior circulation could not be differentiated from the thrombi caused by LAA (TOAST 1) in their composition ratio (*p* = 0.94).

In anterior thrombi there was no statistical difference between TOAST 2 and 5 etiologies (*p* = 0.61), but values of the composition ratio for cardioembolic thrombi (TOAST 2) compared to LAA thrombi (TOAST 1) were significantly lower (*p* = 0.04).

## Discussion

In the present study, cerebral thrombi of patients with BAO were analyzed for thrombus composition. Clear differences were shown compared to thrombus composition of the anterior circulation: (A) basilar thrombi contained an overall higher fraction of RBCs. (B) Basilar thrombi did not have a specific pattern of thrombus composition for each stroke subtype (opposed to anterior thrombi). (C) All basilar thrombi had a similar thrombus composition to LAA thrombi (TOAST 1) of the anterior circulation. These findings are possibly based on different thrombus evolution processes in the posterior circulation with a higher proportion of appositional thrombus growth.

For the first time, to our knowledge, a collective of clots of the posterior circulation was analyzed concerning their histopathological thrombus composition. In the present study, 59 thrombi were gathered between 2008 and 2017. Although this number appears only moderate, this sample size exceeds all previously published histological analyses of occlusions within the posterior circulation by far, which is due to the lower frequency of BAO compared to occlusions in the anterior circulation [10–15].

Previous analyses with very low numbers of BAO occlusions found no significant differences between anterior and posterior circulation thrombi [10]. In our comparison of basilar and anterior thrombi, differences in histopathological composition could be detected. Overall, basilar thrombi showed a higher RBC content. At first sight, it seems plausible that an overall higher RBC amount is due to a higher etiological proportion of LAA in the posterior compared to the anterior circulation, based on a higher number of in situ thromboses [4] or embolism from stenosis of the vertebral artery; however, the higher RBC amount was observed in all other etiological subgroups. Thus, the overall higher RBC amount in the BAO thrombi seems not to be driven by a relatively higher number of patients with LAA stroke but may have pathophysiological reasons.

In patients with LAA stroke, RBC proportions are similar between basilar and anterior thrombi. It seems reasonable that thrombi caused by LAA do not differ between the anterior and posterior circulation, as pathogenesis is similar. Thrombus formation is based on either embolism (e.g. due to a stenosis with ruptured plaque) or local thrombosis. This kind of thrombus evolution is characterized by an acute formation of thrombus, containing platelet aggregations and relatively high amounts of interspersed RBCs.

Most studies on anterior thrombi showed that RBC proportion is higher in thrombi caused by LAA, than in cardioembolic clots [10, 11]. This is different in basilar thrombi with a higher proportion of RBC for all underlying stroke causes, including cardioembolic strokes. This might be attributed to a different thrombus evolution process, as the posterior circulation is characterized by different flow conditions compared to the anterior circulation [16, 18], probably causing a relevant amount of fresh appositional local thrombus with subsequently higher RBC amounts of the thrombi extracted from the posterior circulation.

Importantly, as it is known from experimental models, embolus trajectory may depend on thrombus size and density [23, 24]. The differing diameter of posterior circulation vessels could explain the overall difference of thrombus composition in these thrombi and should be considered in future analysis.

It is assumed that thrombus composition influences the efficacy of different thrombectomy techniques, improving the results of endovascular treatment [25]. The interventionalists could adapt their technique (e.g. utilization of aspiration) also dependent on the expected composition (besides different flow conditions [16]), as RBC thrombi tend to be softer and might be easier to extract with aspiration. The higher RBC count of basilar thrombi would reinforce the primary application of this technique in the vertebrobasilar system. Furthermore, future studies on larger numbers of clots from the posterior circulation could confirm previous studies on anterior circulation occlusions that showed that thrombus composition can be assessed by imaging parameters and could also give valuable information about pathogenesis [26, 27]. This would be valuable in the planning of the endovascular treatment.

Analysis of cerebral thrombi may support decision making concerning secondary prophylaxis after stroke in future [28]; however, as histological differentiation between stroke etiology does not work in basilar thrombi of our study population, our findings clearly show that results of studies focusing on anterior circulation stroke may be transferred to BAO stroke only carefully.

Our study has certain limitations. As thrombi could be gathered for about half of the entire BAO population only, a selection bias cannot be excluded, which affects all studies investigating retrieved thrombi. To evaluate this possible bias, we additionally performed a comparison between the group of patients with evaluable thrombi and the screened patients without analyzable clots regarding demographical, clinical and interventional variables (see supplementary material). This analysis showed no relevant differences in demographic and clinical parameters. There were no differences in the devices used for mechanical recanalization (stand-alone aspiration, stent-retriever only or mixed) and in reperfusion success (measured by mTICI), making a systematic bias unlikely. The longer recanalization time and higher total number of maneuvers within the group of patients without analyzable clots is caused by the outliers of hard clots or clots impossible to remove.

A further limitation is the method of histological analysis. We studied relative quantitative fractions of the different clot components only; however, the clot structure itself was not investigated. To differentiate the parts of appositional thrombus growth with an assumed dominant RBC amount this approach would be helpful and should be applied in further studies. Finally, although TOAST classification was originally designed independently of vascular territory [29], we could not exclude the possibility that the posterior vascular territory affected diagnosis and work-up of stroke etiology in our patients. This, in turn would affect the comparison between subgroups.

## Conclusion

Evidence for a differing thrombus composite was shown between anterior and posterior circulation with an overall higher RBC amount in basilar thrombi. This is possibly based on a different thrombus evolution process in the posterior circulation with a higher proportion of appositional thrombus growth. Results of studies with anterior thrombi, especially regarding the evaluation of secondary prophylaxis strategies, may be transferred to BAO stroke only carefully.

## Abbreviations

BAO: Basilar artery occlusions
F/P: Fibrin/platelets
LAA: Large-artery atherosclerosis
mRS: Modified Rankin scale
MT: Mechanical thrombectomy
mTICI: Modified thrombolysis in cerebral infarction
NIHSS: National Institutes of Health Stroke Scale
RBC: Red blood cells
WBC: White blood cells
